# Antioxidant, Cytotoxicity, Antimicrobial Activity, and *In Silico* Analysis of the Methanolic Leaf and Flower Extracts of *Clitoria ternatea*

**DOI:** 10.1155/2023/8847876

**Published:** 2023-09-22

**Authors:** Md. Ariful Islam, Samiran Kumar Mondal, Shirmin Islam, Most. Nourin Akther Shorna, Suvro Biswas, Md. Salah Uddin, Shahriar Zaman, Md. Abu Saleh

**Affiliations:** ^1^Microbiology Laboratory, Department of Genetic Engineering and Biotechnology, University of Rajshahi, Rajshahi 6205, Bangladesh; ^2^Department of Botany, University of Rajshahi, Rajshahi 6205, Bangladesh

## Abstract

Infectious diseases pose a significant threat to human health worldwide. To address this challenge, we conducted a comprehensive study on the leaf and flower extracts of *Clitoria ternatea* plants. Our research encompassed in vitro assessments of their antibacterial, antibiofilm, antioxidant, and cytotoxic properties. Additionally, we employed in silico screening to identify promising compounds with potential applications in developing novel anti-*Escherichia coli* medications. Notably, our investigation revealed a remarkable inhibition zone of 13.00 ± 1 mm when applying the leaf extract (200 *μ*g/ml) against *E. coli*, showcasing its potent antibacterial properties. Furthermore, both the leaf and flower extracts exhibited substantial biofilm inhibition efficacy against *S. aureus*, with inhibition percentages of 54% and 58%, respectively. In the realm of antioxidant activity, the leaf and flower extracts of *C. ternatea* displayed noteworthy DPPH free radical scavenging capabilities. Specifically, the leaf extract exhibited a substantial activity of 62.39% at a concentration of 150 *μ*g/ml, while the flower extract achieved 44.08% at the same concentration. Our study also evaluated the impact on brine shrimp, where the floral extract displayed a significantly higher mortality rate of 93.33% at a dosage of 200 *μ*g/ml compared to the leaf extract. To elucidate potential therapeutic targets, we utilized molecular docking techniques, focusing on the acbR protein (5ENR) associated with antibiotic resistance in *E. coli*. In this analysis, compounds isolated from the *C. ternatea* leaf extract, namely D1 (CID-14478556), D2 (CID-6423376), and D3 (CID-20393), exhibited binding energies of −8.2 kcal/mol, −6.5 kcal/mol, and −6.3 kcal/mol, respectively. Additionally, compounds from the flower extract, E1 (CID-5282761), E2 (CID-538757), and E3 (CID-536762), displayed binding energies of −5.4 kcal/mol, −5.3 kcal/mol, and −5.1 kcal/mol, respectively. In conclusion, the leaf and flower extracts derived from *C. ternatea* represent a promising natural resource with potential therapeutic applications in combating antibiotic-resistant pathogens.

## 1. Introduction

It is well-established that people and plants have a strong relationship [1]. Humans rely on plants for a variety of purposes, including food, medicine, and domestic use [1]. Plants have always been an essential source of drug invention [2]. In developing countries, approximately 70–80% of the population still relies on the herbal drug for their primary healthcare [3]. Secondary metabolites present in the plant are responsible for beneficial medicinal effects [3].

Conventional herbal medicine is employed globally to treat a wide range of illnesses, such as cancer, diabetes, and heart disease [4]. Plants produce a wide range of chemical compounds known as auxiliary metabolites. Three primary classes of these compounds are terpenes, nitrogen-containing compounds, and phenolic compounds, each possessing unique organic properties that make them valuable in addressing various medical conditions, including cancer, neurological disorders, diabetes, wounds, atherosclerosis, cardiovascular diseases, and injuries [5]. Plant extracts find extensive applications in various industries, including food, cosmetics, and pharmaceuticals, underscoring the importance of conducting systematic research on medicinal plants to explore their therapeutic potential, biological properties, safety profiles, and diverse active compounds [6, 7].

*Clitoria ternatea* belongs to the Fabaceae family and is commonly known as butterfly pea or blue pea flower, with the Bengali name “Aparajita” [8]. This plant is a long-lived climber and is cultivated as an ornamental plant in many countries [8]. There are several species of *C. ternatea* with varying flower colors, including light blue, dark blue, white, and mauve [8]. Blue pea plants are found in several countries worldwide, including Thailand, Malaysia, Kenya, Australia, the USA, Sri Lanka, Brazil, Cuba, Sudan, and others. [9]. In several Southeast Asian countries, blue pea flowers are consumed as a vegetable [10], and extracts from these flowers are used in desserts and beverages [11]. *C. ternatea* serves various agricultural and medicinal purposes, including use as animal feed, nitrogen-fixing crops, an environmentally friendly insecticide, and food coloring and in traditional medicine for conditions such as anasarca . Recent studies have suggested that different parts of *C. ternatea* exhibit sedative properties and antimicrobial, anti-inflammatory, analgesic, antipyretic, and immunomodulatory activities [12].

The misuse and overuse of antibiotics in human and animal healthcare, along with inadequate infection control measures in medical facilities, have given rise to a significant global health concern known as antibiotic resistance [13]. Within the field of biomedical science, there is a strong emphasis on recognizing the value of therapeutic plants [14]. To effectively and economically identify potential drug candidates during the drug discovery process, considerable attention has been given to computer-aided drug design (CADD) methodologies [15]. In particular, in silico methods have proven to be valuable in predicting new drugs and identifying potential targets by utilizing compound structures prior to their actual synthesis [16]. Consequently, the objective of this research was to investigate the antimicrobial properties of methanolic extracts derived from the leaves and flowers of *Clitoria ternatea* in controlled laboratory conditions (in vitro). The selected bacteria for evaluation include *Escherichia coli*, *Salmonella typhi*, *Staphylococcus aureus*, and *Pseudomonas* sp. Additionally, this study aimed to assess the extracts' ability to inhibit the formation of biofilms, evaluate their antioxidant activity using DPPH, determine their cytotoxic effects on *Artemia salina*, and identify potent compounds present in the leaf and flower extracts that specifically target the acrB protein of *E. coli*.

## 2. Materials and Methods

### 2.1. Collection and Preparation of Plant Extracts

Fresh leaves and flowers of *C. ternatea* were harvested from Rajshahi University campus, Bangladesh, and a taxonomist from the Department of Botany, University of Rajshahi, identified the plant. The voucher number of the specimen is denoted by an accession no. 46 and deposited to the herbarium, Department of Botany, University of Rajshahi. These plant parts underwent a process wherein they were finely chopped and then subjected to shade drying. The resultant plant materials were then transformed into a powdered form using a grinder. These powdered plant components were meticulously placed within two separate conical flasks, with an appropriate quantity of solvent added, adhering minimum ratio of 1 : 3 (1 gram of powder to 3 milliliters of solvent). Subsequently, the conical flasks were placed onto an orbital shaker, employing 90 mm Whatman filter paper for extract filtration. This was repeated in triplicate. Following this, the conical flasks were left uncovered for a period spanning from 24 to 48 hours to allow for natural evaporation. The resulting extracts were then concentrated utilizing a rotary evaporator, maintaining a temperature of 40°C. Only the residues were collected and placed into labeled glass vials, which were then stored in a refrigerator at 4°C to ensure preservation. Additionally, the extract was dissolved in dimethyl sulfoxide (DMSO) for the purpose of conducting these experiments.

### 2.2. Antimicrobial Test

Antimicrobial susceptibility testing was performed using the disc diffusion method [17]. Four bacteria (*S. aureus, E. coli, S. typhi,* and *Pseudomonas* sp.) were streaked on agar plate. The sterile filter paper discs were impregnated with known amounts of the test substances and dried and placed on plates. Gentamicin (10 *μ*g/disc) was used as a standard disc. These plates were then kept at a low temperature (4°C) for 24 h to allow maximum diffusion. After that, the plates were kept in an incubator (37°C) for 12−18 h to allow the growth of the organisms. Antimicrobial agents were identified by the formation of zones of inhibition that kill or inhibit microbial growth [18].

### 2.3. Biofilm Formation Assay

The identified strains of bacteria were inoculated into wells of a 96-well microtiter plate (Tarsons, India) containing 100 *μ*l of Luria-Bertani (LB) liquid medium (Himedia, India) and incubated at 37°C for 24 hours. The glass slides were removed from the incubation chamber after the elapsed time, washed twice with double-distilled water, and then oven-dried for an hour at 37°C. The recovered wells were stained with crystal violet (0.1%) to assess biofilm formation. The glass slides were washed twice with double-distilled water and once more after 60 minutes. The OD_595_ was determined using a microplate reader after the slides had been air-dried. Each strain's degree of biofilm development was assessed using OD values [19, 20].

  (A) Nonbiofilm producer (Doa ≤ Docn);
  (B) Weak biofilm producer (Docn < Doa ≤ 2 × Docn);
  (C) Moderate biofilm producer (2 × Docn < Doa ≤4 × Docn); and
  (D) Strong biofilm producer (4 × Docn < Doa)

The experiment was conducted as planned to see if biofilm formation was inhibited. In this case, 100 *μ*l of plant extract was introduced along with the bacteria. The following equation was used to get the inhibition percentage:

(1) $$\begin{array}{c} \text{Inhibition percentage}=\frac{\left({\text{OD}}_{\text{control}}-{\text{OD}}_{\text{sample}}\right)\times 100}{{\text{OD}}_{\text{control}}}\text{.} \end{array}$$

### 2.4. Antioxidant Activity Test

The DPPH (2,2-diphenyl-1-picrylhydrazyl) free radical scavenging assay, with BHT (butylated hydroxytoluene) as a control, was used to assess the antioxidant potential of *C. ternatea* leaf and flower extracts [21]. Four autoclaved test tubes were employed for the DPPH scavenging activity test: one for BHT and three for the extract of *C. ternatea*, each at concentrations of 50, 100, and 150 *μ*g/ml. Then, methanol was added to each test tube to make 1 ml volume. Finally, each test tube received 1 ml of the DPPH solution, and the total volume was 2 ml. To complete the interaction, the test tubes were kept in the dark for thirty minutes at room temperature. Using a spectrophotometer and a reference blank solution, the absorbance of the solutions was measured at 517 nm. To ensure the experiment's accuracy, each absorbance value was repeated three times before the mean absorbance of the solution was determined. The percentage (%) inhibition activity was calculated from the following equation [22]:

(2) $$\begin{array}{c} \%I=\left\{\frac{\left(A_{0}-A_{1}\right)}{A_{0}}\right\}x100\text{,} \end{array}$$

where %*I* is the percentage of inhibition, *A*_0_ is the absorbance of the control, and *A*_1_ is the absorbance of the sample.

### 2.5. Cytotoxicity Test

The cytotoxicity test was conducted on brine shrimp (*Artemia Salina*) nauplii [23, 24]. Brine shrimp nauplii were hatched at ambient temperature in simulated seawater in a beaker. Five test tubes were then prepared with 25, 50, 100, 150, and 200 *μ*g/ml concentrations of the extracts. Subsequently, 10 freshly hatched nauplii *(Artemia salina)* were placed in each test tube, and the tubes were kept at room temperature for 24 hours. Finally, the LC_50_ values of methanolic leaf and flower extracts were determined and recorded.

### 2.6. Molecular Docking Study

#### 2.6.1. Ligand Preparation

For the docking experiment, the chemical compounds obtained through GC-MS analysis of *C. ternatea* [25] were selected as ligands (Tables S1 and S2). These chemical compounds of *C. ternatea* were retrieved from the PubChem database of chemical molecules, and their activities against biological assays were identified through an extensive literature review [26, 27]. The compounds underwent three-dimensional extraction and energy minimization using the Merck molecular force field (MMFF94) to optimize the objective function.

#### 2.6.2. Protein Preparation

Using a protein data bank (PDB ID: 5ENR), the crystalline structure of *E. coli* proteins was obtained. The shape of the protein was refined, and heteroatoms were removed using Discovery Studio software (version 4.5.0) and PyMol software (version 2.4.0). In Swiss PDB Viewer software (version 4.1), the washed proteins were reduced in energy and simplified by employing the Groningen molecular simulation (GROMOS) 431B force field [28]. The quality and geometry of the protein structure were evaluated with the help of the Ramachandran plot analysis.

#### 2.6.3. Molecular Docking

The AutoDock Vina software (version 1.1.2) was used to conduct a molecular docking study to accurately understand the binding kinetics of the target protein and the retrieved compounds of *C. ternatea* [29, 30]. The software was utilized to generate molecular models from the protein's structure. The PDBQT layout was then applied to the ligand molecules. The software was utilized to generate molecular models from the protein's structure. To group and rank the molecules, the most advantageous binding free energy and docking directions within a 2.0 RMSD range were chosen. Molecular docking was successfully performed with an exhaustiveness of 8 and a range of energy modes set at 10 and 20. Ultimately, no bonded interactions between the AutoDock structures were examined using Discovery Studio and PyMol [31].

### 2.7. Statistical Analysis

GraphPad Prism (version 8.4) was used for the analysis and preparation of all figures, in which all values were reported as the mean ± standard error of the mean.

## 3. Results

### 3.1. Antibacterial Activity Test

**Figure 1:**
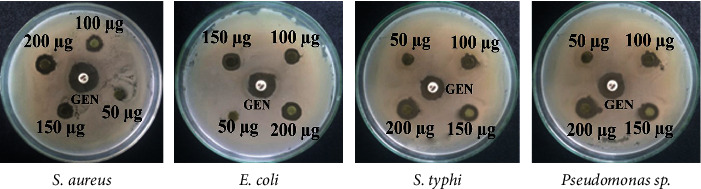
Antibacterial activity of *C. ternatea* leaves extract against *S. aureus*, *(E) coli*, *(S) typhi*, *and Pseudomonas* sp.

**Figure 2:**
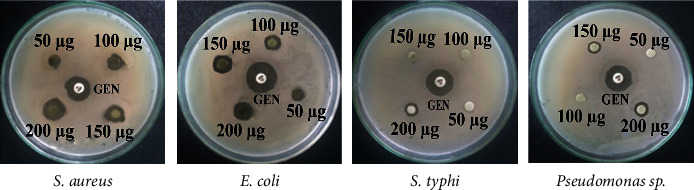
Antibacterial activity of *C. ternatea* flowers extract against *S. aureus*, *(E) coli*, *(S) typhi*, *and Pseudomonas* sp.

The antibacterial activity of *C. ternatea* leaves and flowers extract is shown in Figures 1 and 2. *C. ternatea* leaf extracts showed the highest diameter zone of inhibition against *E. coli* which was 10.33 ± 0.58 mm, 11.00 ± 1 mm, and 13.00 ± 1 mm at the concentration of 100, 150, and 200 *μ*g/ml, respectively (Table 1). The flower extracts of *C. ternatea* showed the highest diameter zone of inhibition against *E. coli,* which was 10.67 ± 0.58 mm, 11.67 ± 0.58 mm, and 12.00 ± 1 mm at the concentration of 100, 150, and 200 *μ*g/ml, respectively (Table 2).

### 3.2. Biofilm Inhibition Assay

**Figure 3:**
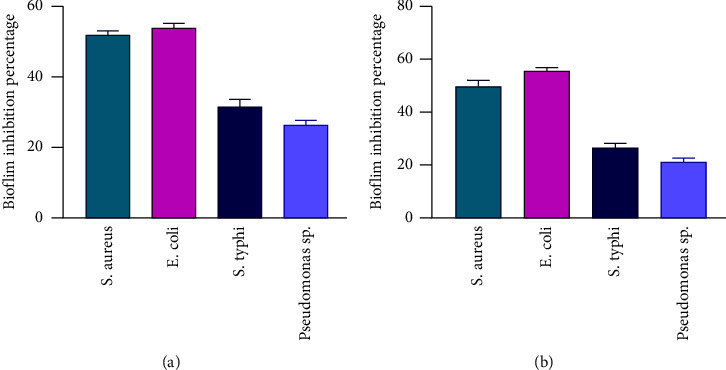
Biofilm forming inhibition assay of *C. ternatea* plants' leaves and flowers extracts. (a) Inhibition assay of leaves extract against four selected bacterial strains and (b) inhibition assay of flowers extract against four selected bacterial strains.

To test the efficacy of *C. ternatea* leaves and flower extracts to prevent the formation of biofilms, four different bacterial strains were used. The four selected bacterial strains had a strong biofilm formation capacity (Table 3). Regarding *S. aureus, E. coli, S. typhi*, and *Pseudomonas* sp., the *C. ternatea* leaf extracts had 52%, 54%, 32%, and 28% of biofilm-forming inhibition efficacy (Figure 3(a)), respectively, while the *C. ternatea* flower extracts had 49%, 58%, 28%, and 21% of biofilm-forming inhibition efficacy, respectively (Figure 3(b)).

### 3.3. Antioxidant Activity Test

**Figure 4:**
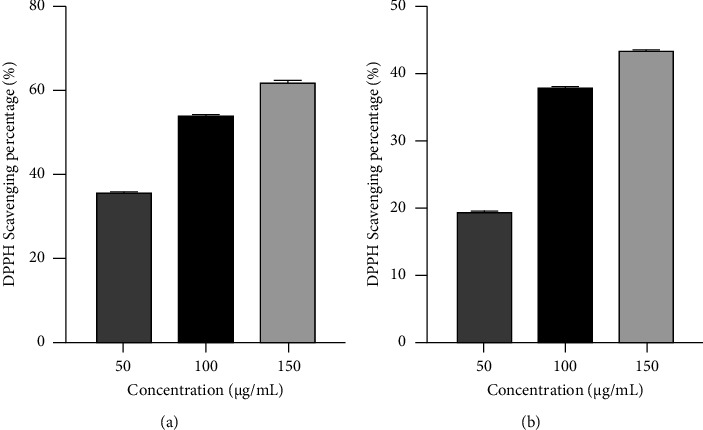
Antioxidant activity of *C. ternatea* plants' leaves and flower extracts. (a) DPPH scavenging activity of leaves extracts and (b) DPPH scavenging activity of flowers extracts.

The antioxidant activity of *C. ternatea* leaf and flower extracts was determined by DPPH free radical scavenging activity. *C. ternatea* leaf extracts showed DPPH free radical scavenging activity of 36.05%, 54.51%, and 62.39% at the concentration of 50, 100, and 150 *μ*g/ml, respectively, with IC_50_ values of 104 *μ*g/ml (Figure 4(a)). On the other hand, *C. ternatea* flower extracts showed DPPH free radical scavenging activity of 19.43%, 38.45%, and 44.08% at the concentration of 50, 100, and 150 *μ*g/ml, respectively, with IC_50_ values of 156 *μ*g/ml (Figure 4(b)).

### 3.4. Cytotoxicity Test

The LC_50_ values of methanolic *C. ternatea* leaf and flower extracts were 62.54 *μ*g/ml and 38.88 *μ*g/ml (Table 4). These findings suggest a strong positive correlation between the concentration of leaf and flower extracts and brine shrimp mortality, with a higher mortality percentage at the 200 *μ*g/ml dose compared to other dosages and a lower mortality percentage at the 50 *μ*g/ml dose. So, at the concentration of 62.54 *μ*g/ml for the leaf extract and 38.88 *μ*g/ml for the flower extract, 50% of brine shrimp could be killed. The percentages of mortality are 40.00 ± 0.58%, 46.66 ± 0.58%, 60.00 ± 0.58%, 73.33 ± 0.58%, and 83.33 ± 1.53% for the *C. ternatea* leaf extracts and 46.66 ± 0.58%, 53.66 ± 0.58%, 66.66 ± 1.53%, 73.33 ± 0.53%, and 93.33 ± 0.58% for the *C. ternatea* flower extracts at the concentrations of 25, 50, 100, 150, and 200 *μ*g/ml (Table 4), respectively.

### 3.5. Molecular Docking Study

**Figure 5:**
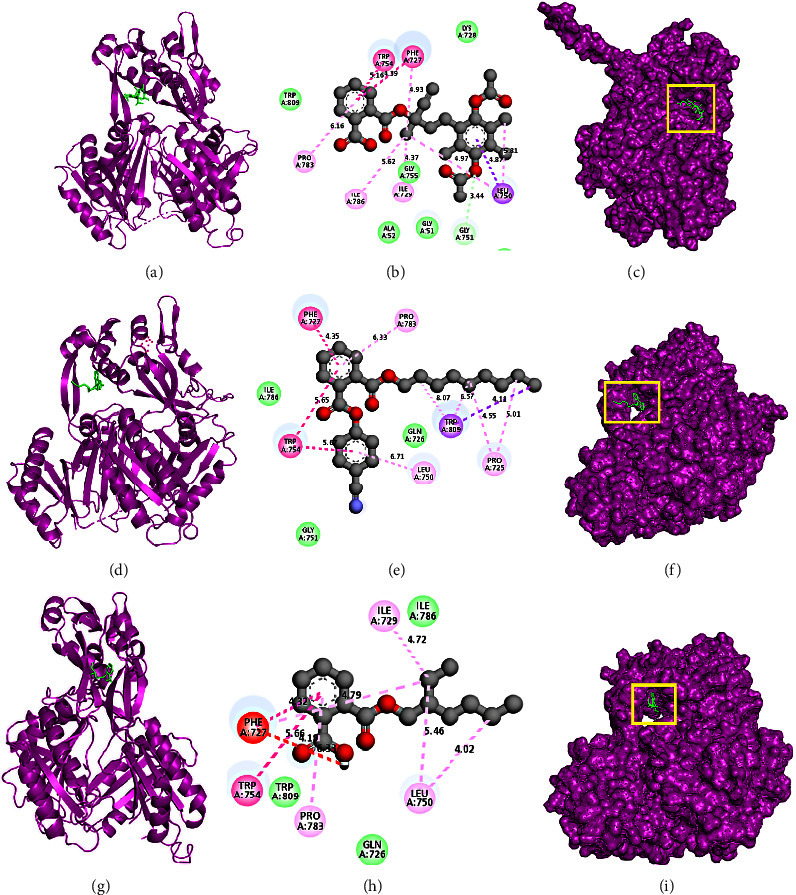
Molecular docking of the acrB protein (5ENR) of *E. coli* and *C. ternatea* leaf chemicals. (a–c) The cartoon view, 2D view, and surface view of the CID-14478556 and acrB protein complex; (d–f) the cartoon view, 2D view, and surface view of the CID-6423376 and acrB protein complex; and (g–i) the cartoon view, 2D view, and surface view of the CID-20393 and acrB protein complex, respectively.

A molecular docking evaluation was conducted to investigate binding interactions and identify principal molecules with a higher affinity for the acrB protein complex. In this study, lower binding scores were obtained for three phytochemicals from *C. ternatea* leaves extract denoting D1, D2, and D3. Here, D1, D2, and D3 refer to CIDs 14478556, 6423376, and 20393, respectively. D1 demonstrated a higher binding affinity (−8.2 kcal/mol) compared to any other substance of *C. ternatea* (Table 5). D1 interacted with *E. coli* protein acrB and formed nine hydrophobic bonds at Phe727, Trp754, Leu750, Pro783, and Ile786 positions. D2 interacted with *E. coli* protein acrB and formed ten hydrophobic bonds at Pro783, Phe727, Trp754, Leu750, Trp809, and Pro725 positions. D3 interacted with *E. coli* protein acrB and formed eight hydrophobic bonds at Ile729, Leu750, Phe727, Trp754, and Pro783 positions (Table 5 and Figure 5).

**Figure 6:**
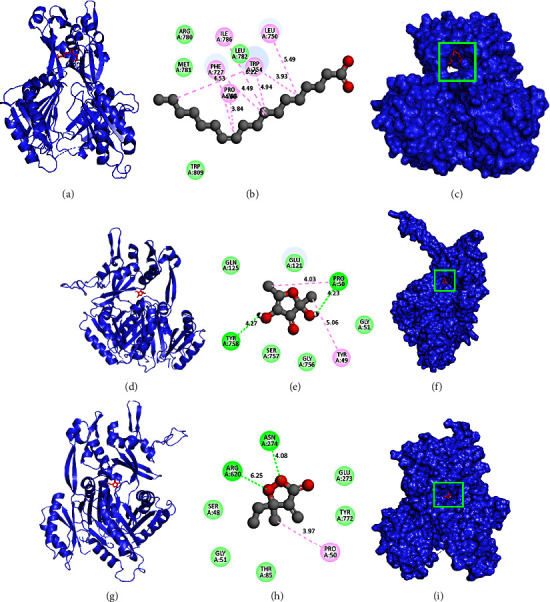
Molecular docking of the acrB protein (5ENR) of *E. coli* and *C. ternatea* flower chemicals. (a–c) The cartoon view, 2D view, and surface view of the CID-5282761 and acrB protein complex; (d–f) the cartoon view, 2D view, and surface view of the CID-538757 and acrB protein complex; and (g–i) the cartoon view, 2D view, and surface view of the CID-536762 and acrB protein complex, respectively.

In the case of flower extracts, lower binding scores were obtained for three phytochemicals from *C. ternatea*, which were marked as E1, E2, and E3, referring to CIDs 5282761, 538757, and 536762, respectively. E1 demonstrated a higher binding affinity (−5.4 kcal/mol) than any other *C. ternatea* flower substance (Table 6). E1 interacted with *E. coli* acrB protein and formed eight hydrophobic bonds at Leu750, Ile786, Trp754, Phe727, and Pro783 positions. E2 interacted with *E. coli* acrB protein and formed two hydrogen bonds at the Pro50 and Tyr758 positions and two hydrophobic bonds at the Pro50 and Tyr49 positions. E3 interacted with *E. coli* acrB protein and formed two hydrogen bonds at Asn274 and Arg620 positions and a hydrophobic bond at Pro50 positions (Table 6 and Figure 6).

## 4. Discussion

Within the field of biological sciences, plants have been utilized as potent sources of substances to control and treat human diseases. Antimicrobial medications are currently used to treat a large number of bacterial and fungal infections. According to Kone et al. [32], bacteria and fungi are known to cause opportunistic diseases. Antibiotic resistance has emerged in many virulently pathogenic microbial species as a result of widespread and indiscriminate usage of antibacterial drugs [32]. Many antimicrobials now in use have negative side effects such as toxicity, hypersensitivity, immunosuppression, and tissue residues, constituting a public health risk. These restrictions reduce the therapeutic efficacy of currently available antibiotics, encouraging the search for alternative therapies for the treatment of bacterial and fungal infections. As the ecosystem shifts toward nontoxic and environmentally friendly products, there is a growing need to prioritize the development of contemporary pharmaceuticals derived from traditional medicinal plants. These can be used for the treatment of a variety of human and animal diseases. *C. ternatea* is a plant known for its various therapeutic characteristics. The indigenous medicine uses several parts of *C*. *ternatea* plant, including the leaves, roots, stems, and flowers, to cure a wide variety of human afflictions. The purpose of the current study was to use *C. ternatea* leaf and flower extracts to control four distinct bacterial strains (*S. aureus, E. coli, S. typhi, and Pseudomonas sp*). We found that *C. ternatea* leaf extracts formed a significant zone of inhibition against *E. coli*, with a diameter of 13 mm. The inhibition zones for the standard Gentamicin (10 *μ*g/disc) ranged from 15 mm to 20 mm against four selected bacteria for the above-mentioned extracts. Upon examination, it was observed that the leaf and flower extracts of *C. ternatea* displayed intermediate resistance against all four selected bacteria, primarily at concentrations of 150 *μ*g/ml and 200 *μ*g/ml. In contrast, they exhibited higher resistance at concentrations of 50 *μ*g/ml and 100 *μ*g/ml against the same bacteria. Based on the data presented, the study concluded that the leaf and flower extracts of *C. ternatea* have antibacterial properties against the tested bacteria. As a result, the expected dose (150 *μ*g/ml or 200 *μ*g/ml) was sufficient to slow down bacterial development, while *C. ternatea* leaf extract was the most effective against the bacteria. These findings corroborate earlier research findings wherein the leaf extracts exhibited comparable antibacterial efficacy against *S. aureus* (11 mm), *E. coli* (13.3 mm), *Pseudomonas* sp. (13.3 mm), and *S. typhi* (12.7 mm), while the flower extracts demonstrated effectiveness against *S. aureus* (11 mm), *E. coli* (13.33 mm), *Pseudomonas* sp. (11.3 mm), and *S. typhi* (10.3 mm). [33].

This study suggests that both leaf and flower extracts of *C. ternatea* have strong abilities to inhibit biofilm formation, which aligns with previous research findings [34]. We found that the extract of *C. ternatea* leaves and flowers exhibited significant antioxidant activity, peaking at 62.39% and 44.08%, respectively, at a concentration of 150 *μ*g/ml. The majority of diseases and ailments are caused by oxidative stress, which is generated by free radicals [35]. Antioxidants have been shown to protect cells from oxidative damage caused by free radicals, which may help to avoid diseases such as cancer and aging. By interacting with free radicals, chelating metals, and serving as oxygen scavengers, they can disrupt the oxidation process [36]. Some of these are alcohol, tobacco, prescription drugs, smoked and barbecued food, pesticides, insecticides, harmful agrochemicals, additives in the foods we eat, and pollutants in the air we breathe [37]. Several studies have shown that synthetic antioxidants such as butylated hydroxytoluene and butylated hydroxyanisole are suspected of being carcinogenic. As a result, antioxidants of natural origin have become a hot topic among modern researchers [38]. In this study, the highest DPPH scavenging activity (62.39%) was observed in *C. ternatea* leaf extracts at a concentration of 150 *μ*g/ml, while the lowest (19.43%) was found in *C. ternatea* flower extracts at 50 *μ*g/ml. The leaf and flower extracts exhibited lower antioxidant activity compared to the standard BHT. Specifically, the leaf extracts showed relatively higher antioxidant activity than the flower extracts. In a study by Fu et al. [39], the antioxidant properties of *C. ternatea's* flower extract were investigated in vitro. The DPPH results for their methanol extract (58%) were found to be lower compared to the methanolic extract used in the current study. Similarly, Jadhav et al. [40] also evaluated the antioxidant properties of *C. ternatea's* flower extract through in vitro testing. Their methanol extract yielded DPPH results (52%) that were slightly higher than those obtained from the methanolic extract utilized in the present study. The Brine shrimp assay is a vital tool in biology, efficiently assessing the toxicity of various substances. It is a swift and cost-effective method for examining chemicals, natural compounds, and environmental samples for potential harm to organisms. Its applications span drug discovery, environmental monitoring, phytochemical research, and initial safety assessments [41–43]. The leaf extract concentration and brine shrimp mortality were positively correlated, as indicated by the LC_50_ values of *C. ternatea's* leaf and flower extracts. Our findings confirmed that the mortality percentages for the leaf and flower extracts were 83.33% and 93.33%, respectively. Rahman et al. [44] performed an in-vitro evaluation to assess the cytotoxic effects of *C. ternatea's* leaf extracts. The cytotoxicity test results from their methanol extract (95%) were similar compared to the methanolic extract utilized in the current study.

Utilizing molecular docking, the binding configuration of two interacting molecules with known structures is identified. This process predicts how the receptor and ligand should ideally align to form a stable complex [20]. The evaluation of docking studies is an effective approach to drug development [45]. Utilizing molecular docking, we assessed the effects of phytochemicals from *C. ternatea* plants on target proteins. From a range of datasets, molecular docking and molecular dynamics investigations can aid in the identification of efficient inhibitors. Our understanding of ligand-protein interactions and target binding affinity is improved by this research [46]. Furthermore, extensive research has been conducted to discover potent inhibitors of target proteins, which is essential for advancing computer modeling and investigating the precise dynamics of ligand-protein interactions [47]. Chian et al. suggest that successful ligand candidates can be distinguished through docking simulations [48]. By intercepting at a protein's active region, the targeted protein can be blocked. 10 chemicals (Table S1) were retrieved from the GC-MS analysis of methanolic leaf extracts, and 15 chemicals (Table S2) were retrieved from the GC-MS analysis of methanolic flower extracts of *C. ternatea* [25, 49]. Our research findings were paired with an in silico technique to identify a possible component in leaf and flower extracts from *C. ternatea* that might be utilized to create medications against *E. coli*. The most potent compounds against the *E. coli* acrB protein (5ENR) were found in *C. ternatea's* leaf extracts, including 1,2-benzenedicarboxylic acid, bis (2-methylpropyl) ester, phthalic acid, 4-cyanophenyl nonyl ester, and 2-([(2-ethylhexyl) oxy] carbonyl) benzoic acid (Table 5). Similarly, the most potent compounds against *the E. coli* acrB protein (5ENR) were identified in *C. ternatea's* flower extracts, which included 2,4-dihydroxy-2,5-dimethyl-3(2H)-furan-3-one, 1,2-dioxolan-3-one, 5-ethyl-5-methyl-4-methylene, and acetic acid, 1-(2-methyltetrazol-5-yl)ethenyl ester (Table 6). 5ENR is a transport protein of *E. coli* that binds to the structure of bacterial efflux pumps, and these pumps are crucial antibacterial drug development targets because efflux pumps play a significant role in multidrug resistance (MDR) [50]. Therefore, compounds derived from both the leaf and flower of the *C. ternatea* plant have the potential to inhibit the receptor domain of the E. coli acrB protein (5ENR).

The findings of this study represent a notable improvement compared to previous research, as we demonstrate the significant antimicrobial properties of methanolic extracts obtained from *C. ternatea* leaves and flowers against a broader range of bacteria, such as *Escherichia coli*, *Salmonella typhi*, *Staphylococcus aureus*, and *Pseudomonas* sp. These extracts also exhibited strong inhibitory effects on biofilm formation and displayed potent antioxidant activity. Furthermore, the extracts demonstrated cytotoxicity towards *Artemia salina*. Through in silico analysis, several potential bioactive compounds including flavonoids, anthocyanins, and phenolic acids were identified in the extracts. These findings suggest that *C. ternatea* holds promise as a valuable source of novel natural products possessing antimicrobial properties. However, further investigations are necessary to identify the active compounds within the extracts and assess their effectiveness in vivo.

## 5. Conclusion

The emergence of new disease-causing pathogens and the development of antibiotic resistance pose significant concerns for the healthcare industry. In this study, methanolic extracts of *C. ternatea* leaves and flowers exhibited significant antibacterial activity. The leaf had the highest antibacterial activity against the four bacterial strains. Although the antioxidant activity of both leaves and flowers was lower than that of the BHT standard, the leaf exhibited higher antioxidant activity compared to the flower. Therefore, the methanolic extract of *C. ternatea* leaves and flowers holds potential as a valuable source of natural antioxidant and antibacterial compounds, offering opportunities for the development of novel pharmaceuticals to combat various human diseases. The flower extracts exhibited higher toxicity to brine shrimp than the leaf extracts, as indicated by the cytotoxicity results. Two compounds, i.e., CID 14478556 from the leaf and CID 5282761 from the flower, displayed higher binding affinities in the active region of the acrB protein (5ENR) during molecular docking tests. Thus, these two could be used for future drug development against *E. coli* infections.

## Figures and Tables

**Table 1:** Antibacterial activity of *C. ternatea* leaf extracts against four selected bacteria.

Name of bacteria	Concentration (*μ*g/ml)	Zone of inhibition (mm)	Result
*S. aureus*	50	8.00 ± 1.00	R
	100	10.00 ± 1.00	I
	150	10.67 ± 0.58	I
	200	12.00 ± 1.00	I

*E. coli*	50	8.33 ± 0.58	R
	100	10.33 ± 0.58	I
	150	11.00 ± 1.00	I
	200	13.00 ± 1.00	I

*S. typhi*	50	8.33 ± 0.58	R
	100	10.00 ± 1.00	I
	150	11.33 ± 0.58	I
	200	11.67 ± 0.58	I

*Pseudomonas* sp.	50	7.00 ± 0.82	R
	100	8.33 ± 1.00	R
	150	10.33 ± 0.58	I
	200	11.67 ± 0.58	I

Note: Zone size <10 mm = resistance (R), zone size 10–15 mm = intermediate resistance (I), and zone size >15 mm = sensitive (S).

**Table 2:** Antibacterial activity of *C. ternatea* flower extracts against four selected bacteria.

Name of bacteria	Concentration (*μ*g/ml)	Zone of inhibition (mm)	Result
*S. aureus*	50	8.33 ± 0.58	R
	100	9.33 ± 0.58	R
	150	11.00 ± 1.00	I
	200	11.67 ± 0.58	I

*E. coli*	50	8.33 ± 0.58	R
	100	10.67 ± 0.58	I
	150	12.33 ± 0.58	I
	200	13.00 ± 1.00	I

*S. typhi*	50	5.67 ± 0.58	R
	100	7.67 ± 0.58	I
	150	9.67 ± 0.58	I
	200	10.67 ± 0.58	I

*Pseudomonas* sp.	50	6.00 ± 1.00	R
	100	7.33 ± 0.58	R
	150	8.67 ± 0.58	R
	200	11.00 ± 1.00	I

Note: Zone size <10 mm = resistance (R), zone size 10–15 mm = intermediate resistance (I), and zone size >15 mm = sensitive (S).

**Table 3:** The biofilm formation efficacy of the selected bacterial strains.

Name of bacteria	Range	Result
*S. aureus*	OD > 4 × ODcutoff	Strong
*E. coli*	OD > 4 × ODcutoff	Strong
*S. typhi*	OD > 4 × ODcutoff	Strong
*Pseudomonas* sp.	OD > 4 × ODcutoff	Strong

**Table 4:** Cytotoxicity activity of *C. ternatea* leaves and flowers extract and LC_50_ values.

Test samples	Con. (*μ*g/ml)	Mortality %	LC_50_
*C. ternatea* leaf	25	40.00 ± 0.58	62.54
	50	46.66 ± 0.58	
	100	60.00 ± 0.58	
	150	73.33 ± 0.58	
	200	83.33 ± 1.53	

*C. ternatea* flower	25	46.66 ± 0.58	38.88
	50	53.33 ± 0.58	
	100	66.66 ± 1.53	
	150	73.33 ± 1.53	
	200	93.33 ± 0.58	

**Table 5:** Noncovalent connections of *C. ternatea* leaf compounds with specific proteins as well as their binding connections by hydrogen and hydrophobic bonds.

CID	Amino acid residues	Bond types	Distance (Å)	Docking score (kcal/mol)
14478556	PHE727	Hydrophobic	4.93	−8.2 kcal/mol
	PHE727	Hydrophobic	4.39	
	TRP754	Hydrophobic	5.16	
	LEU750	Hydrophobic	4.87	
	LEU750	Hydrophobic	4.97	
	LEU750	Hydrophobic	5.81	
	PRO783	Hydrophobic	6.16	
	ILE729	Hydrophobic	4.37	
	ILE786	Hydrophobic	5.62	

6423376	PRO783	Hydrophobic	6.63	−6.5 kcal/mol
	PHE727	Hydrophobic	4.35	
	TRP754	Hydrophobic	5.65	
	TRP754	Hydrophobic	5.67	
	LEU750	Hydrophobic	6.71	
	TRP809	Hydrophobic	4.11	
	TRP809	Hydrophobic	6.57	
	TRP809	Hydrophobic	8.07	
	PRO725	Hydrophobic	4.55	
	PRO725	Hydrophobic	5.01	

20393	ILE729	Hydrophobic	4.72	−6.3 kcal/mol
	LEU750	Hydrophobic	4.02	
	LEU750	Hydrophobic	5.46	
	PHE727	Hydrophobic	4.32	
	PHE727	Hydrophobic	4.19	
	PHE727	Hydrophobic	4.79	
	TRP754	Hydrophobic	5.66	
	PRO783	Hydrophobic	6.33	

The relationship distance was calculated in Å.

**Table 6:** Noncovalent connections of *C. ternatea* flower compounds with specific proteins as well as their binding connections by hydrogen and hydrophobic bonds.

CID	Amino acid residues	Bond types	Distance (Å)	Docking score (kcal/mol)
5282761	LEU750	Hydrophobic	5.49	−5.4 kcal/mol
	ILE786	Hydrophobic	6.22	
	TRP754	Hydrophobic	3.93	
	TRP754	Hydrophobic	4.49	
	TRP754	Hydrophobic	4.53	
	PHE727	Hydrophobic	4.49	
	PHE727	Hydrophobic	4.66	
	PRO783	Hydrophobic	3.84	

538757	PRO50	Hydrogen	4.23	−5.3 kcal/mol
	TYR758	Hydrogen	4.27	
	PRO50	Hydrophobic	4.03	
	TYR49	Hydrophobic	5.06	

536762	ASN274	Hydrogen	4.08	−5.1 kcal/mol
	ARG620	Hydrogen	6.25	
	PRO50	Hydrophobic	3.97	

The relationship distance was calculated in Å.

## Data Availability

The datasets used and/or analyzed during the current study are available from the corresponding author on reasonable request.

## Supplementary Materials

Supplementary Materials
*Table S1.* Compounds present in methanolic leaf extract C. ternatea as well as their CID. No. and docking score. *Table S2.* Compounds present in methanolic flower extract C. ternatea as well as their CID. No. and docking score.
