# Supplementary material for: Antioxidant, Cytotoxicity, Antimicrobial Activity, and In Silico Analysis of the Methanolic Leaf and Flower Extracts of Clitoria ternatea
Source: Biochem Res Int. 2023 Sep 22;2023:8847876. doi: 10.1155/2023/8847876 (PMC10541305; doi:10.1155/2023/8847876)
Supplement: Supplementary Materials — Table S1. Compounds present in methanolic leaf extract C. ternatea as well as their CID. No. and docking score. Table S2. Compounds present in methanolic flower extract C. ternatea as well as their CID. No. and docking score. [file 8847876.f1.docx]

**Antioxidant, cytotoxicity, antimicrobial activity, and *In silico* analysis of the methanolic leaf and flower extracts of *[Clitoria ternatea](https://en.wikipedia.org/wiki/Clitoria_ternatea)***

**Md. Ariful Islam^1,#^, Samiran Kumar Mondal^1,#^, Shirmin Islam^1^, Most. Nourin Akther Shorna^2^, Suvro Biswas^1^, Md. Salah Uddin^1^, Shahriar Zaman^1^, Md. Abu Saleh^1,*^**

^1^Microbiology Laboratory, Department of Genetic Engineering and Biotechnology, University of Rajshahi, Rajshahi- 6205, Bangladesh.

^2^Department of Botany, University of Rajshahi, Rajshahi-6205, Bangladesh.

# Indicates equal contribution

*Correspondence to: [saleh@ru.ac.bd](mailto:saleh@ru.ac.bd)

Table S1: Compounds present in methanolic leaf extract *C. ternatea* as well as their CID. No. and docking score.

| S/N | Name of Compound | CID. No. | Docking score |
| --- | --- | --- | --- |
| 1 | Dodecanoic Acid, Methyl Ester | 8139 | -5.2 |
| 2 | 1,2-Benzenedicarboxylic Acid, Bis (2- Methylpropyl) Ester | 14478556 | -8.2 |
| 3 | 1,2-Benzenedicarboxylic Acid, Dibutyl Ester | 3026 | -5.6 |
| 4 | 1,2-Benzenedicarboxylic Acid, Bis(2- Methylpropyl) Ester | 6782 | -6.2 |
| 5 | 2-([(2-Ethylhexyl) Oxy] Carbonyl) Benzoic Acid | 20393 | -6.3 |
| 6 | 1-Penten-4-Yn-3-Ol, 1-Chloro-3-Ethyl | 5281077 | -4.3 |
| 7 | Phthalic Acid, 4-Cyanophenyl Nonyl Ester | 6423376 | -6.5 |
| 8 | 1,2-Benzenedicarboxylic Acid, Mono (2- Ethylhexyl) Ester | 6426665 | -6.1 |
| 9 | 1,2-Benzenedicarboxylic Acid, Dipentyl Ester | 8561 | -5.7 |
| 10 | 1,2-Benzenedicarboxylic Acid, Butyl Octyl Ester | 66540 | -5.5 |

Table S2: Compounds present in methanolic flower extract *C. ternatea* as well as their CID. No. and docking score.

| S/N | Name of Compound | CID. No. | Docking score |
| --- | --- | --- | --- |
| 1 | Glycerin | 753 | -4.1 |
| 2 | 2,4-Dihydroxy-2,5-dimethyl-3(2H)-furan-3-one | 538757 | -5.3 |
| 3 | 2-Hydroxy-gamma-butyrolactone | 545831 | -4.2 |
| 4 | Acetic acid, 1-(2-methyltetrazol-5-yl)ethenyl ester | 538650 | -5.0 |
| 5 | dl-Glyceraldehyde dimer | 533979 | -4.1 |
| 6 | Alpha-amino-gamma-butyrolactone | 73509 | -4.0 |
| 8 | 1,2,3-Propanetriol, monoacetate | 33510 | -4.3 |
| 9 | 1,2-Dioxolan-3-one, 5-ethyl-5-methyl-4- methylene | 536762 | -5.1 |
| 10 | 4-Ethylamino-n-butylamine | 7567962 | -3.7 |
| 12 | 2-Propenoic acid, tridecyl ester | 18316 | -4.9 |
| 13 | Neophytadiene | 10446 | -4.9 |
| 14 | n-Hexadecanoic acid | 985 | -5.1 |
| 15 | cis-Vaccenic acid | 5282761 | -5.4 |
